# MAPK Signaling Drives Inflammation in LPS-Stimulated Cardiomyocytes: The Route of Crosstalk to G-Protein-Coupled Receptors

**DOI:** 10.1371/journal.pone.0050071

**Published:** 2012-11-30

**Authors:** W. Joshua Frazier, Jianjing Xue, Wendy A. Luce, Yusen Liu

**Affiliations:** 1 Department of Pediatrics, The Ohio State University College of Medicine, Columbus, Ohio, United States of America; 2 Center for Perinatal Research, The Research Institute at Nationwide Children's Hospital, Columbus, Ohio, United States of America; 3 Center for Cardiovascular and Pulmonary Research, The Research Institute at Nationwide Children's Hospital, Columbus, Ohio, United States of America; Wayne State University School of Medicine, United States of America

## Abstract

Profound cardiovascular dysfunction is an important cause of mortality from septic shock. The molecular underpinnings of cardiac dysfunction during the inflammatory surge of early sepsis are not fully understood. MAPKs are important signal transducers mediating inflammation whereas G-protein signaling pathways modulate the cardiac response to stress. Using H9c2 cardiomyocytes, we investigated the interaction of MAPK and G-protein signaling in a sepsis model to test the hypothesis that the cardiomyocyte inflammatory response is controlled by MAPKs via G-protein-mediated events. We found that LPS stimulated proinflammatory cytokine production was markedly exacerbated by siRNA knockdown of the MAPK negative regulator Mkp-1. Cytokine production was blunted when cells were treated with p38 inhibitor. Two important cellular signaling molecules typically regulated by G-protein-coupled receptors, cAMP and PKC activity, were also stimulated by LPS and inflammatory cytokines TNF-α and IL-6, through a process regulated by Mkp-1 and p38. Interestingly, neutralizing antibodies against Gα_s_ and Gα_q_ blocked the increase in cellular cAMP and PKC activation, respectively, in response to inflammatory stimuli, indicating a critical role of G-protein coupled receptors in this process. LPS stimulation increased COX-2 in H9c2 cells, which also express prostaglandin receptors. Blockade of G-protein-coupled EP4 prostaglandin receptor by AH 23848 prevented LPS-induced cAMP increase. These data implicate MAPKs and G-proteins in the cardiomyocyte inflammatory response to LPS as well as crosstalk via COX-2-generated PGE_2_. These data add to our understanding of the pathogenesis of septic shock and have the potential to guide the selection of future therapeutics.

## Introduction

Septic shock is the most severe manifestation of systemic infection and is a major cause of morbidity and mortality worldwide [1]. In the United States approximately 750,000 patients are treated for severe sepsis yearly with a mortality rate of 30–50% and an estimated $17 billion in health care costs [1], [2]. Despite advances in diagnosis, antibiotic therapy and supportive care, mortality has remained high and disproportionately affects the chronically ill and the aged [1], [2].

A key feature of septic shock, particularly in the early stage, is the severe and often dynamic changes that adversely affect cardiovascular performance which ultimately impair delivery of oxygen to tissues [3], [4]. Preclinical studies as well as investigations of septic patients have led to the conclusion that sepsis-related cardiovascular dysfunction is a highly complex and multifactorial disease process [5]. Various inputs, such as pathogen-specific factors, host immunity, and baseline cardiovascular status, all contribute to the shock phenotype. Moreover, hemodynamic perturbations in septic shock vary depending on stage of the disease and in response to resuscitative measures [6], [7]. Developmental differences in cardiovascular physiology and systemic inflammation exist such that septic shock presents (and is treated) differently in the very young [6], [8]. These highly variable aspects of septic shock have driven investigators to examine the molecular events which underlie septic disease in order to better understand pathogenesis and formulate therapy.

A robust body of literature supports the notion that cytokines and other proinflammatory mediators produced in response to invasive infection have profound effects on cardiovascular function. Such effects are adaptive when short-lived, for example increased capillary permeability which delivers host leukocytes to the site of infection. Septic shock however represents a state of disordered cytokine production in response to systemic inflammation [3], [4]. In this environment, cytokine-mediated impairments in contractility, capillary permeability and vasomotor tone are highly detrimental in that they result in mismatch between oxygen supply and demand at the cellular level. During invasive infection, innate immune effector cells such as monocytes and macrophages are the first-line defenders and are implicated as the source of early proinflammatory cytokine production [9]. Control of cytokine production is in these cells is governed by signal transduction systems which communicate extracellular stimuli to the host cell nucleus and mediate the host response. We have previously investigated the role of the MAPK system in the host response to inflammation [10], [11], [12], [13], [14], [15], [16]. In several models of systemic inflammation, including clinically-relevant murine sepsis, we have demonstrated that MAPKs are key mediators driving the production of inflammatory cytokines during sepsis [10], [12], [13]. Additionally, we have established the regulatory phosphatase Mkp-1 as a crucial regulator of MAPK activity which plays a vital role in down-regulating cytokine production and restraining inflammation [10], [11], [12], [13], [14], [15], [16]. A complimentary and intensely studied signal transduction system involves the action of guanine nucleotide-binding (G) proteins, which are activated after stimulation of G-protein-coupled receptors (GPCRs) [17]. G-proteins exist as heterotrimers which dissociate after stimulation of their GPCR. Activated G-protein subunits then then affect the generation of second messenger molecules and a host of cellular responses, including inflammation [17], [18]. Although considerable data support the importance of both MAPK and G-protein signaling in models of sepsis and other inflammatory processes [19], [20], [21], [22], the importance of these pathways and their crosstalk in the pathogenesis of septic shock-related cardiovascular dysfunction is not completely understood.

Using an in vitro system with H9c2 cardiomyocytes, we examined MAPK, Mkp-1, and G-protein-coupled cellular signaling events to test the hypothesis that MAPKs play a key role in the cardiomyocyte response during sepsis, and that MAPK and G-protein pathways crosstalk to mediate the complex cellular processes during the inflammatory response in these heart cells.

## Materials and Methods

### Reagents

*Mkp1* siRNA was synthesized by Thermo Scientific (Rockford, IL). Antibodies against the N-terminus of Gα_s_ and Gα_q_, p38 inhibitor VIII (referred as ED1428), the EP4 antagonist AH 23848, and the TNF-α inhibitor CAY10500 were purchased from Santa Cruz Biotechnology (Santa Cruz, CA). The EP2 antagonist AH 6809 was purchased from Cayman Chemical Company (Ann Arbor, MI). LPS, IL-6, TNF-α, and streptolysin O were purchased from Sigma Chemicals (St Louis, MO). PCR primers were synthesized by Integrated DNA Technologies Inc. (Coralville, IA). cAMP was measured using a cAMP EIA kit from Cayman Chemical Company. Primer sequences for the PCR experiments are presented in Table 1.

**Table 1:** Primer sequences.

Gene	Sequence	Product
MKP-1 forward	5′-CTA CCA GTA CAA GAG CAT CCC-3′	210 bp
MKP-1 reverse	5′-AAC TCA AAG GCC TCG TCC AG-3′	
RPL30 forward	5′-GAT CAG ACA AGG CAA AGC GA-3′	189 bp
RPL30 reverse	5′-TCA ATG ATA GCC AGT GTG CA-3′	
IL-6 forward	5′-GAA ATG AGA AAA GAG TTG TGC A-3′	147 bp
IL-6 reverse	5′-TTT TCA ATA GGC AAA TTT CC-3′	
TNF-α forward	5′-CTC CCA GGT TCT CTT CAA GG-3′	280 bp
TNF-α reverse	5′-CTC CAA AGT AGA CCT GCC C-3′	
EP1 forward	5′-GAA CTC TAA CTC CCT GCA GC-3′	184 bp
EP1 reverse	5′-CAG GCA CTC TTG GTT AGG C-3′	
EP2 forward	5′-CAC GAT GCT CAT GCT CTT C-3′	236 bp
EP2 reverse	5′-GCG TAC AGC TGA AGG TAT GC-3′	
EP3 forward	5′-GTC ATC CTC GTG TAC CTG TC-3′	200 bp
EP3 reverse	5′-CGA GTC TTC ATG TGG CTG G	
EP4 forward	5′-CTG GTG GTG CTC ATC TGC TC-3′	220 bp
EP4 reverse	5′-CCA ATG CGG CAG AAG AGG C-3′	
DP forward	5′-CTT CAT GGT ACC TCT GGC C-3′	159 bp
DP reverse	5′-CAG ACT GAA GAT GTG GTA GG-3′	

### Cell culture

Monolayers of H9c2 cells (American Type Culture Collection, Manassas, VA) were maintained in high glucose DMEM (Invitrogen), supplemented with 10% FCS (Hyclone, Logan, UT), 100 IU/mL penicillin and 100 µg/mL streptomycin in humidified atmosphere of 5% CO_2_ at 37°C. Cells were trypsinized and seeded at a density of 3×10^5^ cells per well in 12-well culture plates. Cells were grown 48 h to achieve confluence before experiments.

### siRNA transfection

H9c2 cells (6×10^4^ cells per well) were seeded in 12-well plates, incubated 24 h, and then transfected with either 50 nM scramble RNA (SC, as control) or 50 nM *Mkp1* siRNA for 6 h at 37°C, using 1 µl/mL DharmaFECT Duo (Thermo Scientific). The transfection medium was then switched to standard medium, and cells were allowed to recover. Cells were subsequently stimulated on the next day. Supernatants were collected for determining the concentration of cytokines. Transfection efficiency was assessed by transfecting cells with a fluorescent form of Lamin A/C siRNA (siGLO Lamin A/C Control siRNA) which enables correlation of fluorescence uptake with silencing activity.

### Cytokines

TNF-α, IL-6, and IL-1β concentrations in the medium were determined using ELISA kits from BD Biosciences (San Diego, CA), according to the manufacturer's instructions.

### Cell permeabilization with streptolysin O

Confluent H9c2 cells in 12-well culture plates were washed twice with 0.5 mL permeabilizing buffer containing 5 mM HEPES (pH 7.3), 120 mM KCl, 25 mM NaHCO_3_, 10 mM MgCl_2_, 1 mM KH_2_PO_4_, 1 mM EGTA, and 100 µM ATP. Cell membrane permeabilization was carried out by incubation with 20 U/mL streptolysin O in 0.5 mL permeabilizing buffer for 5 min, followed by two washes with 0.5 mL permeabilizing buffer. The permeabilization buffer was then changed to standard medium and the cells were incubated overnight with antibody.

### Detection of camp

H9c2 cells in 12-well culture plates were washed twice with 0.5 mL EBSB buffer containing 1× Earle's balanced salt solution (Sigma Chemicals), 10 mM HEPES (pH 7.4), 2 mM glutamine, 25 mM NaHCO_3_, and 1 mg/mL BSA. Cells were pre-incubated for 20 min in EBSB buffer at 37°C, then incubated with 100 µM Ro-20-1724 (a potent cAMP-specific phosphodiesterase inhibitor) for 30 min at 37°C. Samples were assayed for cAMP using an EIA kit according to manufacturer's instructions.

### Detection of PKC activity

Confluent H9c2 cells in 12-well culture plates were washed twice with 0.5 mL ice-cold PBS, and then lysed in lysis buffer (20 mM MOPS (pH 7.4), 50 mM β-glycerophosphate, 50 mM NaF, 1 mM Na_3_VO_4_, 5 mM EGTA, 1% NP40, 1 mM DTT, 1 mM benzamidine, 1 mM PMSF, 2 µg/mL leupeptin, and 2 µg/mL aprotinin) for 30 min at 4°C. Cells were scraped, sonicated, and the lysates centrifuged. The activity of PKC kinase in the soluble cell lysates was measured using a kit purchased from Assay Designs (Ann Arbor, MI), according to the manufacturer's recommendations.

### Isolation of RNA

H9c2 cells were denatured with 4 M guanidine thiocyanate, mixed with 2 M sodium acetate (pH 4.0), and subsequently extracted with phenol, phenol/chloroform/isoamyl alcohol, and finally precipitated with isopropanol. RNA pellets were washed with 75% ethanol and dissolved in DEPC water. The quality of the total RNA was analyzed by agarose/formaldehyde gel electrophoresis, and the integrity of the RNA samples was indicated by the presence of intact 28S and 18S bands.

### Reverse transcriptase real-time PCR

RNA was reverse-transcribed by using MuLV reverse transcriptase (Applied Biosystems) and oligo dT primer. Real-time PCR was performed in an iCycler (Bio-Rad, Hercules, CA) using iQ SYBR Green Supermix qPCR Kit (Bio-Rad) with the following program: 3 min 95°C denaturation, 40 thermal cycles composed of a 30 sec denaturation step at 95°C, 10 sec annealing step at 60°C, and 30 sec extension step at 72°C. Real-time PCR results were normalized to the housekeeping gene ribosomal protein L30 (RPL30).

### Western blots

Soluble lysate protein (20 µg) was resolved using 4–12% NuPAGE Bis-Tris gel (Invitrogen), and transferred to PVDF membranes (Bio-Rad). Membranes were blocked, probed with antibodies against Mkp-1 (Santa Cruz Biotechnology), p-p38 (Cell Signaling Technology), p38 (Santa Cruz Biotechnology), ERK (Cell Signaling Technology), and β-Actin (Sigma Chemicals). The membranes were incubated with horseradish peroxidase-conjugated secondary antibodies (1∶15,000) for 30 min at room temperature. Immunoreactive bands were visualized with the ECL kit (GE Healthcare, Piscataway, NJ).

## Results

### Mkp-1 plays an important role in the regulation of the inflammatory response in cardiomyocytes

**Figure 1:**
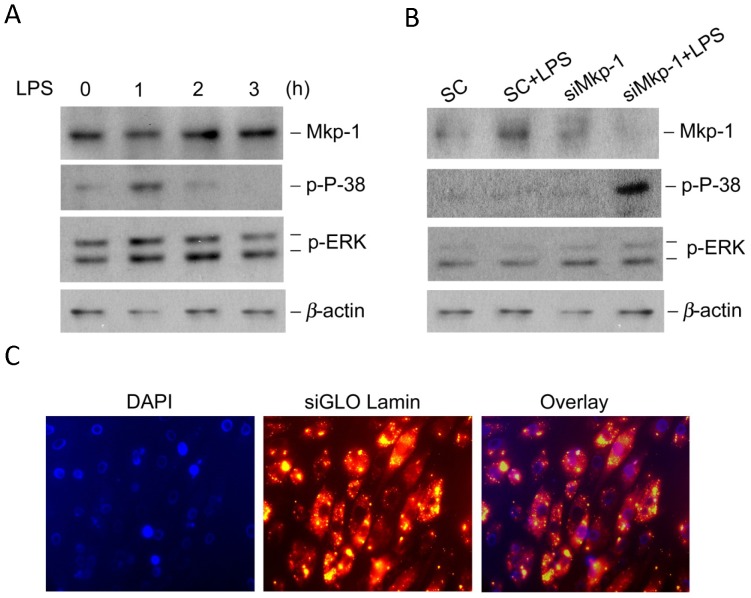
LPS transiently activates MAP kinases in H9c2 cardiomyocytes. (*A*) The kinetics of p38 and ERK activation and Mkp-1 induction in response to LPS stimulation. H9c2 cardiomyocytes were stimulated with LPS (5 µg/ml) for indicated periods of time prior to harvest harvested. Levels of Mkp-1, phospho-38 and phospho-ERK were assessed by Western blot. The membrane was stripped and reblotted with an antibody against β-actin to verify comparable loading. (*B*) The effects of Mkp-1 knockdown on p38 and ERK activity in LPS-stimulated H9c2 cells. H9c2 cells were transiently transfected with either siRNA of *Mkp1* (si*Mkp1*) or scrambled RNA (SC). Twenty-four h after transfection, the cells were stimulated with LPS for an additional 18 h. Cell lysates were subjected to Western blot. (*C*) Verification of siRNA transfection by fluorescent microscopy. H9c2 cells were transiently transfected with fluorescent siRNA of Lamin A/C. After 48 h, cells were fixed, stained with DAPI, and subjected to fluorescent microscopy. Note that nuclei were stained blue and cytosolic compartments red due to the uptake of DY-547-labeled Lamin siRNA. Transfection efficiency was estimated to be >90%. Images presented are representative results (40× magnification).

To understand the mechanisms involved in myocardial dysfunction during sepsis, we investigated the roles of MAPK signaling pathways in the regulation of inflammation in H9c2 rat cardiomyocytes. LPS stimulation of cultured H9c2 cardiomyocytes led to a time-dependent activation of p38 and ERK MAPKs. Increases in the levels of phospho-p38 and phospho-ERK were detectable 1 h post stimulation. Mkp-1, a negative regulator of MAPKs, was present throughout the course of the studies but increased above basal levels at 2 h. Increased Mkp-1 was temporally associated with diminished phospho-p38 and, to a much lesser extent, phospho-ERK (Figure 1A). These findings suggest that Mkp-1 may act as a negative regulator of MAPKs in H9c2 cardiomyocytes as has been established previously in several models of inflammation using immune effector cells [12], [13], [15], [23].

To test the hypothesis that Mkp-1 regulates cytokine production in cardiomyocytes during inflammation in a manner similar to innate immune cells, H9c2 cells were transfected with either scrambled RNA, marked as SC in the figures, or *Mkp1*-interfering siRNA (designated as siMkp-1) to knockdown *Mkp-1* (Figure 1B). As expected, transfection with si*Mkp1* resulted in substantially decreased Mkp-1 protein level following LPS stimulation and markedly increased levels of phospho-p38. The transfection efficiency was demonstrated using a fluorescent form of Lamin A/C siRNA (siGLO Lamin A/C Control siRNA) (Figure 1C). In the transfected cells, the nuclei were stained with DAPI, while the cytosol compartments were seen as red due to uptake of DY-547-labeled Lamin siRNA. Transfection efficiency was >90%.

**Figure 2:**
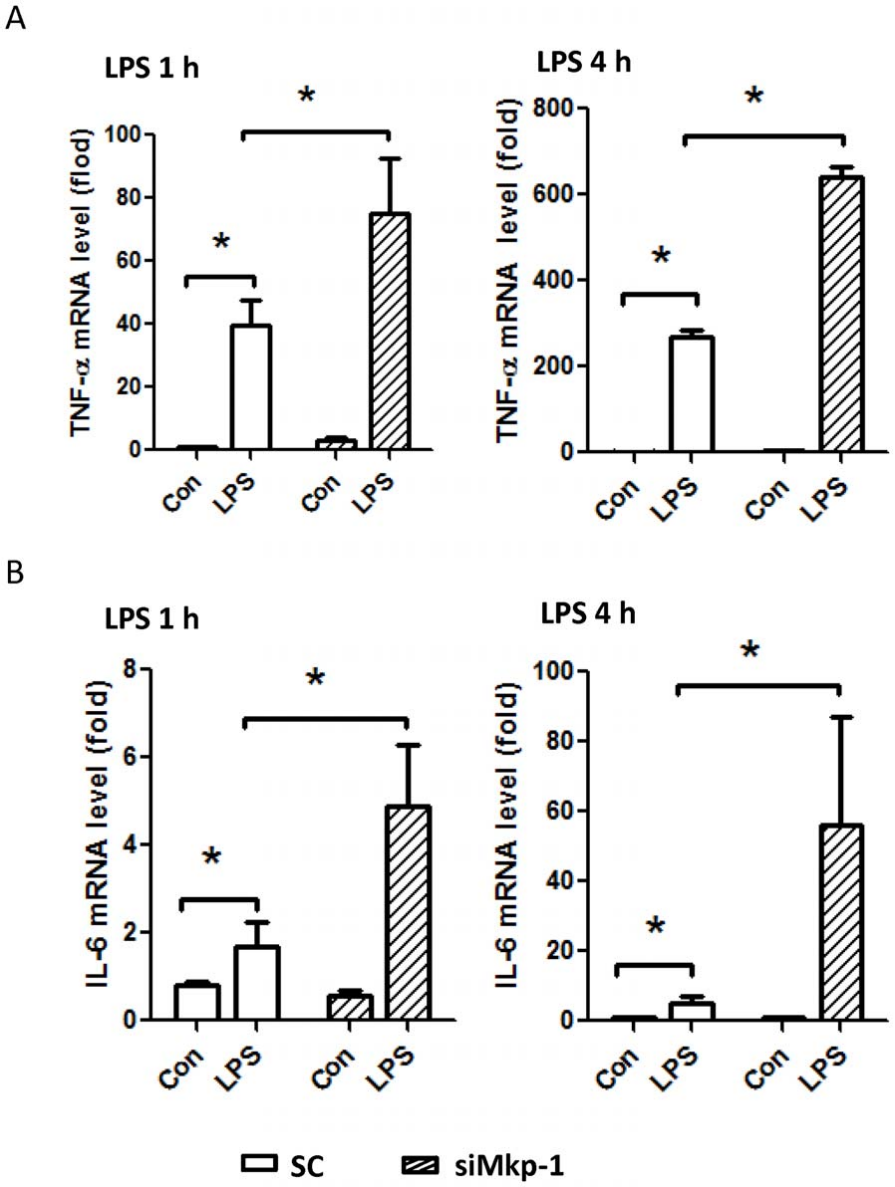
Mkp-1 negatively regulates cytokine expression in LPS-stimulated H9c2 cardiomyocytes. H9c2 cells were transfected with *Mkp1* siRNA or a scrambled RNA control (SC). After 48 h, cells were stimulated with 5 µg/mL LPS for either 1 or 4 h. Total RNA was harvested, and cDNA synthesized using reverse transcriptase. Expression levels of TNF-α (*A*) and IL-6 (*B*) were assessed by qPCR. The expression of TNF-α and IL-6 were normalized to the housekeeping gene RP30, and presented as fold change relative to scramble RNA-transfected, unstimulated cells. Data are means ± SE from at least 3 independent experiments. *, *P*<0.05.

**Figure 3:**
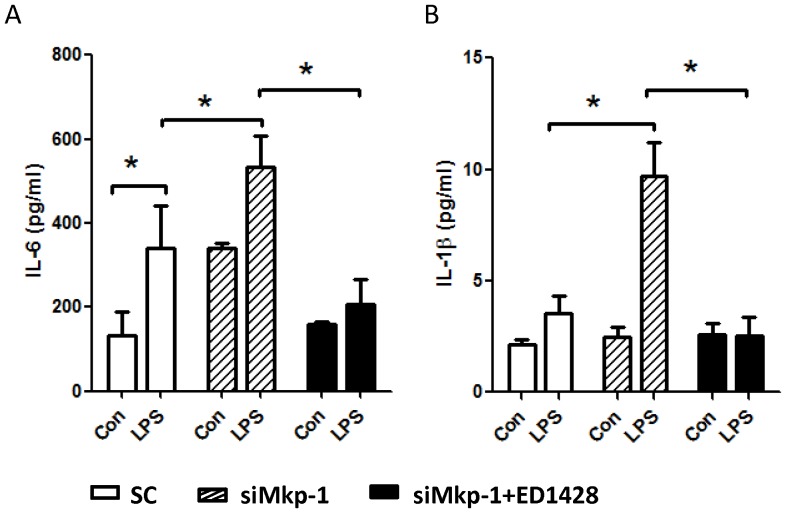
The production of IL-1β and IL-6 in response to LPS stimulation is positively regulated by p38 and negatively controlled by Mkp-1 in cardiomyocytes. H9c2 cells were transfected with *Mkp1* siRNA or a scrambled RNA control (SC). Twenty-four h after transfection, the cells were pretreated with either DMSO, as control (Con), or with the p38 inhibitor ED1428 for 30 min, and subsequently stimulated with 5 µg/mL LPS for 18 h. The concentrations of IL-6 (*A*) and IL-1β (*B*) in the culture medium were determined by ELISA. Data are presented as means ± SE from at least 3 independent experiments. *, *P*<0.05.

To investigate the impact of *Mkp1* knockdown on the inflammatory response in H9c2 cardiomyocytes, we assessed the effect of LPS stimulation on inflammatory cytokine production. Following LPS stimulation, *Mkp1* knockdown cells produced significantly higher levels of mRNA transcripts for the proinflammatory cytokines TNF-α and IL-6 after both 1 h and 4 h exposures compared to cells transfected with scramble RNA (Figure 2). With regard to cytokine protein production, the effects of *Mkp1* knockdown were in keeping with the mRNA results. LPS-stimulated production of IL-6 and IL-1β by H9c2 cells was significantly increased after pretreatment with si*Mkp1* (Figure 3). Interestingly, treatment with a potent p38 inhibitor, ED1428 [24], abolished the effects of si*Mkp1* on IL-1β and IL-6 protein production, with levels of each being similar to unstimulated cells (Figure 3). These data support the conclusion that LPS-induced cytokine production in H9c2 cardiomyocytes is mediated by p38, and in turn negatively regulated by Mkp-1.

### The p38 MAPK regulates PKC activation and cAMP production in LPS-stimulated cardiomyocytes

**Figure 4:**
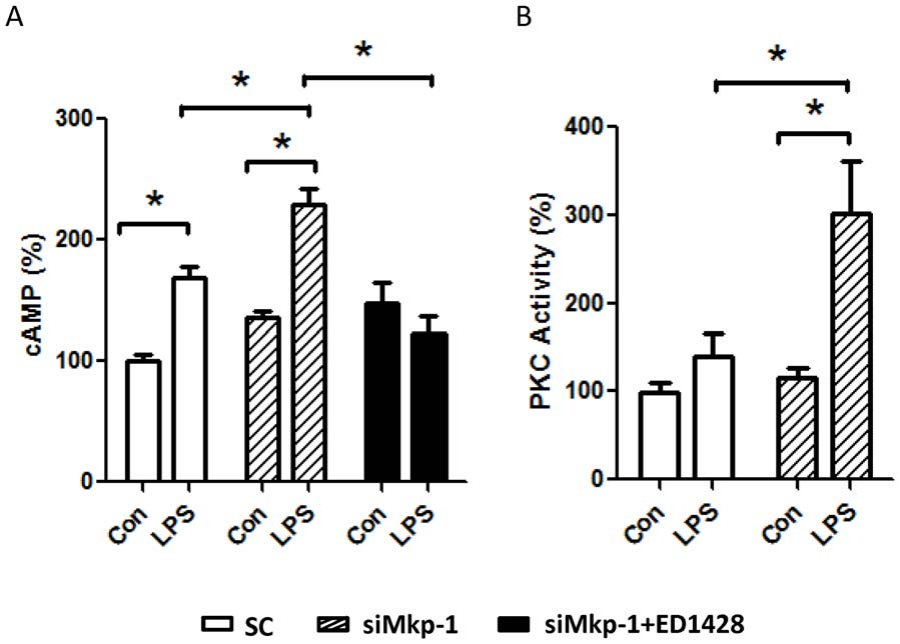
LPS stimulation leads to increased levels of cellular cAMP and PKC activity in H9c2 cardiomyocytes. H9c2 cells were transfected with either *Mkp1* siRNA or a scrambled RNA (SC) as control. Twenty-four h after transfection, cells were pretreated with either DMSO (Con) or with the p38 inhibitor ED1428 for 30 min, and subsequently stimulated with 5 µg/mL LPS for 18 h. Cells were harvested and cAMP levels were assessed using an EIA kit (*A*). PKC activity in cell lysates was assessed using a non-radioactive colorimetric PKC kinase activity kit (*B*). Results are expressed as % relative to scramble RNA-transfected cells that neither received p38 inhibitor nor were stimulated with LPS. Data represent means ± SE from at least 5 independent experiments, *<0.05.

LPS-induced acute cardiac failure during sepsis is associated with alterations in G-protein-mediated signal transduction [25]. To understand whether G-protein-mediated cell signaling is controlled by MAPK and Mkp-1 during LPS challenge, we investigated the roles of p38 and Mkp-1 on G-protein-mediated subcellular events. G-proteins are important signal transducers which generate second-messenger molecules involved in a wide variety of cellular responses, including innate immune responses [17], [18]. Two key effector systems operating downstream of the GPCRs are the adenylate cyclase/cAMP and phospholipase C (PLC)/protein kinase C (PKC) pathways. As the result of stimulation of GPCRs, Gα_s_ subunits activate the adenylate cyclase, leading to cAMP production, while Gα_q_ activates PLC-β, which in turns causes activation of the classic PKCs [18]. We found that stimulation of H9c2 cells with LPS resulted in a significant increase in the level of cellular cAMP (Figure 4). Importantly, transfection with siMkp-1 prior to LPS stimulation led to markedly higher levels of cAMP compared to transfection with scramble control RNA. In contrast, pretreatment of H9c2 cardiomyocytes with p38 inhibitor ED1428 prior to LPS challenge attenuated the LPS-induced increase in cAMP, suggesting an important role of p38 in the regulation of the cAMP second messenger (Figure 4A). We also examined the effect of LPS stimulation and the role of Mkp-1 on PKC activity in cardiomyocytes (Figure 4B). In H9c2 cells transfected with scramble control RNA, LPS stimulation resulted in a ∼50% increase in PKC activity. However *Mkp1* knockdown with si*Mkp1* led to a nearly three-fold increase in PKC activity in LPS-stimulated cardiomyocytes.

### The role of Gα_s_, Gα_q_, and prostaglandin receptors in the regulation of cAMP and PKC in LPS-stimulated cardiomyocytes

**Figure 5:**
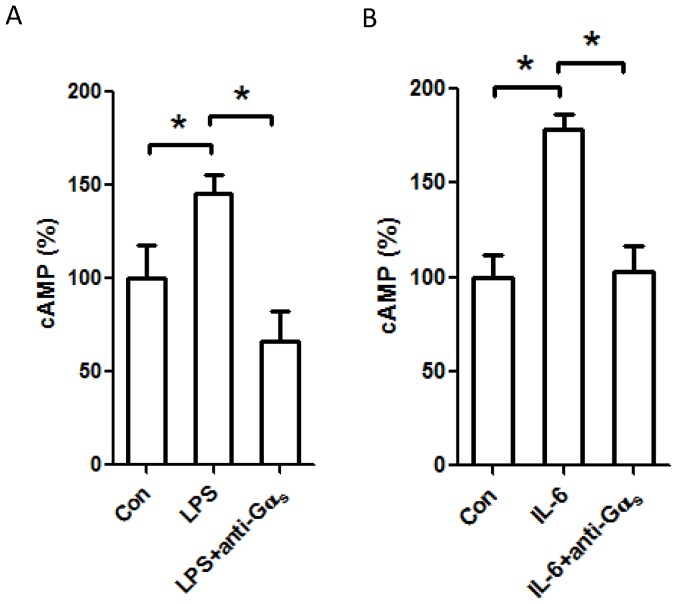
Increases in intracellular cAMP following LPS and exogenous IL-6 stimulation are mediated via by Gα_s_. H9c2 cells were permeablized with an antibody against Gα_s_, or left untreated, and then stimulated with LPS (*A*) or IL-6 (*B*) for 18 h. Cells were harvested, and levels of intracellular cAMP were assessed using an EIA kit. Data represent means ± SE of at least 3 experiments. *, *P*<0.05.

To address the role of G-protein signaling in cardiomyocytes during the inflammatory response, we neutralized Gα_s_ and Gα_q_ subunits with specific antibodies and examined the effects on downstream signaling molecules. Both Gα_s_ and Gα_q_ associate with GPCRs to initiate signal transduction following binding to theirs cognate ligands [18]. While control antibody did not affect the increase in cellular cAMP in response to LPS stimulation (data not shown), anti-Gα_s_ antibody significantly decreased the cAMP level (Figure 5A). In fact, in Gα_s_ neutralizing antibody-treated cells cAMP levels dropped to levels below what was observed in unstimulated cells, even after LPS stimulation. Additionally, Gα_s_ neutralizing antibody not only prevented cAMP increase in response to LPS, but also reduced cAMP in response to IL-6 stimulation (Figure 5B).

**Figure 6:**
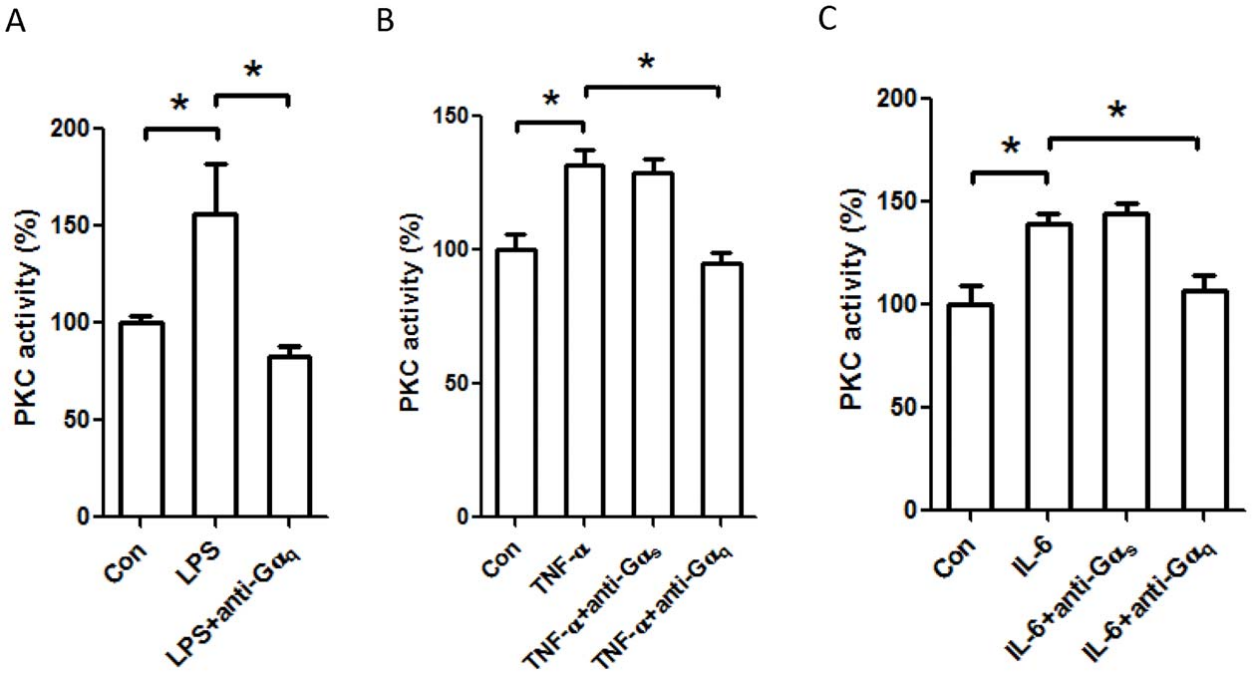
Blockade of Gα_q_, but not Gα_s_, with a neutralizing antibody abolishes PKC activation in response to inflammatory stimuli in H9c2 cardiomyocytes. H9c2 cells were permeablized with antibodies against either Gα_s_ or Gα_q_ and then stimulated with LPS (*A*), or TNF-α (*B*), or IL-6 (*C*) for 18 h. Cells were harvested, and levels of PKC activity in the cell lysates were assessed using a nonradioactive colorimetric PKC activity assay kit. Results are expressed as % relative to the unstimulated control cells. Data represent mean ± SE of at least 3 experiments. *, *P*<0.05.

We also assessed the role of G-protein in PKC activation in H9c2 cells following exposure to inflammatory stimuli. Neutralizing Gα_q_ antibody abolished PKC activation induced by LPS, suggesting an important role of Gα_q_ in PKC activation during the cardiac inflammatory response (Figure 6A). Gα_q_ neutralization also abrogated PKC activation in response to stimulation by inflammatory cytokines. PKC activation in response to TNF-α (Figure 6b) and IL-6 (Figure 6C) was prevented by an antibody against Gα_q_. Interestingly, Gα_s_ neutralizing antibody did not influence TNF-α- or IL-6-induced PKC activity (Figure 6B and C).

**Figure 7:**
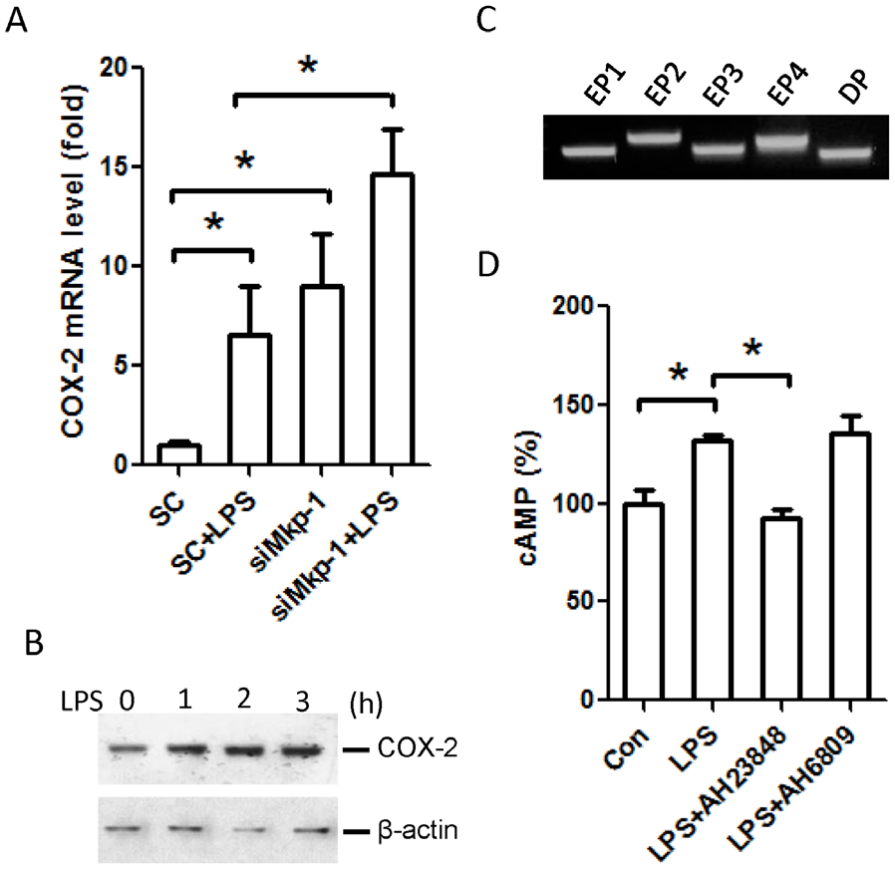
PGE_2_ receptor EP4 plays an important role in mediating increased intracellular cAMP during the cardiomyocyte response to LPS. (*A*) The effects of *Mkp1* knockdown on COX-2 mRNA induction by LPS. H9c2 cardiomyocytes were transfected with either scrambled RNA (SC) or *Mkp1* siRNA. After 48 h, cells were stimulated with LPS (5 µg/mL) for 4 h or left unstimulated. Total RNA was harvested and COX-2 expression was assessed by PCR. COX-2 expression is presented as fold change relative to unstimulated SC controls. (*B*) The effects of LPS on COX-2 protein expression. H9c2 cells were stimulated with LPS (5 µg/mL) for the indicated periods of time. COX-2 protein in lysates was assessed by Western blot. The membrane was stripped and reblotted with a β-actin antibody to verify comparable protein loading. (*C*) Detection of the mRNA transcripts of different prostaglandin receptors in H9c2 cardiomyocytes. Total RNA was extracted from unstimulated H9c2 cells. The transcripts of the receptors of PGE_2_ (EP1–4) and PGD_2_ (DP) were amplified by RT-PCR. PCR products were separated by electrophoresis on a 2% agarose gel. (*D*) The differential effects of prostaglandin receptor antagonists on LPS-induced increases in cAMP. H9c2 cells were first pretreated with DMSO (as control (Con)), 10 µM AH23848 (an inhibitor of EP4), or 10 µM AH 6809 (inhibitor of EP1, EP2, EP3, and DP) for 60 min, and then stimulated with LPS for an additional 18 h. Cells were harvested and intracellular cAMP levels were determined using an EIA kit. Data represent means ± SE of at least 3 experiments. *, *P*<0.05. Images shown in panels *B* and *C* are representative results.

Prostaglandins are important mediators in the inflammatory response in many cell types, and account for many of the physiological changes observed during inflammation [26], [27]. COX-2 is highly induced during the inflammatory reaction, and its products, prostaglandins, can exert their biological activities through G-protein-coupled receptors [27], [28]. For example, PGE_2_ can interact with four distinct receptors (EP1–4) to modulate downstream biological pathways via activated G-proteins [28]. While PGE_2_ interaction with EP1 activates Gα_q_, PGE_2_ interaction with either EP2 or EP4 activates Gα_s_. Since COX-2 is a gene induced by inflammatory stimuli in many cell types [29], we examined the effects of LPS on COX-2 expression in cardiomyocytes. As indicated in Figure 7A, LPS robustly enhanced the expression of COX-2 in H9c2 cells. Western blot analysis indicated that COX-2 protein levels were enhanced in response to LPS stimulation in H9c2 cells (Figure 7B). Using RT-PCR we confirmed that all four PGE_2_ receptors, EP1, EP2, EP3, and EP4, as well as the PGD_2_ receptor, DP, are expressed in H9c2 cells (Figure 7C).

To determine whether prostaglandins are involved in the cellular events typically controlled by the G-protein-coupled receptors in H9c2 cardiomyocytes, we blocked PGE_2_ receptors using antagonists, and assessed the effects on cellular cAMP after LPS stimulation. Interestingly, the specific EP4 antagonist AH23848 abrogated the increase in cAMP in LPS-stimulated cells (Figure 7D), while the antagonist AH6809, which blocks EP1, EP2, EP3, and DP, had no effect. These results clearly indicate that prostaglandins produced by COX-2 contribute to the physiological changes in cardiomyocytes during the inflammatory response.

## Discussion

### MAPKs and Mkp-1 in cardiomyocyte inflammatory response

We have previously reported on the importance of MAPK restraint by Mkp-1 in the host inflammatory response to a variety of systemic inflammatory challenges. Using *Mkp-1^−/−^* mice we have shown that Mkp-1 is necessary for control of cytokine production following challenge with LPS and peptidoglycan [12], [13], [15]. *Mkp-1^−/−^* mice exposed to LPS or infected with live *E. coli* suffer accelerated and greater overall mortality from shock compared to wild type controls [10]. This shock state is characterized by marked elevations of TNF-α and IL-6 and biochemical and histological evidence of profound cardiovascular impairment [10]. Further detail regarding cardiac performance was provided by Zhang et al. who documented echocardiographic evidence of severely altered heart function associated with prolonged MAPK activation and elevated myocardial TNF-α in *Mkp-1^−/−^* mice after LPS challenge [23]. In this current work, we employed H9c2 cardiomyocyte cells and modified MAPK signaling to measure changes in cardiomyocyte-specific inflammatory events. In our system, LPS exposure resulted in a time-dependent increase in activated p38 and ERK MAPKs. Subsequently, increased Mkp-1 was temporally associated with reduction in active MAPK levels (Figure 1). When assayed for cytokine production, cardiomyocytes demonstrated robust production of TNF-α and IL-6, in terms of both mRNA and protein, at 1 h and 4 h after LPS (Figures 2 and 3). These kinetics in H9c2 cells closely mirror data from previous investigations of innate immune effector cells and coincide with prior reports establishing an intrinsic inflammatory capability of cardiomyocytes that does not require the influence of circulating leukocytes or their mediators [23], [30]. Just as in innate immune cells, p38 plays a crucial role in the production of both TNF-α and IL-6 in H9c2 cardiomyocytes (Figure 3). Control of the H9c2 inflammatory response was also heavily influenced by the functional status of Mkp-1. Knockdown of *Mkp1* with siRNA greatly enhanced the inflammatory phenotype, both in terms of proinflammatory p38 activity (Figure 1B) and the resultant inflammatory cytokine production (Figures 2 & 3). These data establish MAPK signaling as an important pathway for cardiomyocyte response to LPS, with Mkp-1 serving as a restraining mechanism for this inflammatory response. This notion is further strengthened by the finding that p38 inhibitor ED1428 abrogated the production of both IL-1β and IL-6 triggered by LPS. Our in vitro data, coupled with the in vivo and physiologic data of others [23], [25], [31], [32], [33] bring to light intriguing possibilities regarding the molecular underpinnings of cardiovascular dysfunction in sepsis. Currently a focus of intense research, myocardial dysfunction during sepsis represents an important cause of morbidity and mortality. Although MAPKs, especially p38, are important in the development of myocardial dysfunction [31], [34], [35] including myocardial infarction [36] and cardiomyopathy [37], [38] the role of MAPK regulation in sepsis-induced cardiovascular dysfunction is not fully understood. In addition, other signal transduction pathways influence the cardiovascular response to inflammation. Crosstalk between pathways is therefore an important area for investigation [20], [21], [22], [37], [39], [40], [41], [42].

### Mkp-1 controls G-protein-mediated events in cardiomyocytes

Among the most studied of various signal transduction pathways important in inflammation, G-proteins are known to modulate a vast array of cellular processes, including leukocyte trafficking in response to chemotactic agents [43], [44], [45]. G-proteins become activated when their associated GPCRs interact with the cognate ligands [18]. Upon GPCR stimulation, Gα subunits bind GTP and dissociate from the Gβγ subunit. Both subunits are biologically active and affect a number of events depending on subtype. Pertinent to our current discussion, the stimulatory Gα_s_ subunit activates adenylate cyclase, leading to production of cAMP while Gα_q_ activates PKC [18]. cAMP is a potent second messenger molecule which activates PKA and affects cardiovascular performance. In previous reports, it has been shown that both experimental sepsis and adrenergic stimulation lead to increased myocardial cAMP via G-protein stimulation; Wu et al. found that late-stage sepsis is associated with increases in inhibitory G-proteins (Gα_i_) and depressed cardiac function [33]. In our H9c2 cells we found that two signaling mediators typically regulated by GPCRs, intracellular cAMP and PKC, were modulated by inflammatory stimuli. Cellular cAMP levels were elevated in response to LPS and IL-6 in a Gα_s_-dependent manner (Figure 5). Furthermore, PKC activity was enhanced in response to LPS, TNF-α, and IL-6 in a Gα_q_-dependent manner (Figure 6). We documented significant increases in cAMP and PKC activity after LPS and IL-6 that were further exacerbated by si*Mkp1*. Conversely, the cellular increase in cAMP in response to LPS was significantly attenuated by p38 inhibition. These data establish a role for p38 MAPK in influencing the G-protein pathway in cardiomyocytes. Previous investigators have demonstrated multilevel crosstalk between the MAPK and G-protein signaling systems [19], [21], [22], [40]. G-proteins can influence ERK activity at multiple levels [19]. Moreover, both p38 and JNK can be regulated by G-proteins [40]. Here, we provide evidence of crosstalk between G-proteins and MAPK in an opposite direction, with MAPK activating G-proteins in cardiomyocytes. Since the receptors for LPS, TNF-α, and IL-6 are not classic GPCRs, G-protein-mediated cellular events, such as cAMP increases and PKC activation (Figure 4), are likely triggered by a common downstream process during the inflammatory response.

### COX-2 and prostaglandins in MAPK/G-protein crosstalk

While our data provide strong evidence supporting the involvement of GPCRs during the cardiomyocyte response to inflammatory challenges, the exact mechanisms involved remain unclear, especially as they pertain to MAPK/G-protein crosstalk. One potential intermediary process driving the increased cellular cAMP and PKC activation after the inflammatory stimuli, which are not sensed by GPCRs, is enhanced MAPK-driven prostaglandin production and subsequent activation of intracellular prostaglandin receptors [28]. Prostaglandins are produced by cyclooxygenases, particularly COX-2, in response to inflammatory stimuli. In previous investigations we have found increased COX-2 activity in *Mkp-1^−/−^* animals during experimental sepsis compared to wild type controls [10]. Most arachidonic acid metabolites produced by COX-2, including prostaglandin (PG) E_2_, function through intracellular GPCRs. We postulate that GPCR-regulated events (e.g. increased cAMP and PKC) in H9c2 cells exposed to non-GPCR inflammatory stimuli are at least partly mediated by prostaglandins in H9c2. Our results support this conclusion in the following manner: *1*) as demonstrated in Figure 7A and B, COX-2 is potently induced by LPS; *2*) LPS-induced COX-2 expression is positively regulated by p38 and negatively regulated by Mkp-1; *3*) Prostaglandins exert their cellular activities via GPCRs which regulate cAMP and PKC; *4*) The GPCR PGE_2_ and PGD_2_ receptors are expressed in H9c2 cardiomyocytes (Figure 7C); and *5*) Blockade of EP4 with the antagonist AH23848 abolishes LPS-induced cAMP elevation in H9c2 cardiomyocytes (Figure 7D). Interestingly, while the other prostaglandin GPCRs EP1, EP2, EP3, and DP are also expressed in H9c2 cardiomyocytes, the antagonist AH6809 had no effect on cellular cAMP induced by LPS treatment (Figure 7D). It is possible that in H9c2 cells the expression levels of the other PGE_2_ or PGD2 receptors are relatively low, despite the fact that their expression was detected by our highly sensitive RT-PCR assay. It should be noted that activation of GPCRs is a late event in the complex cardiomyocyte responses to inflammatory stimuli. The unraveling of EP4 in the regulation of G-protein-mediated cellular processes during the cardiomyocyte inflammatory response likely only represents the tip of an iceberg. While it is plausible that other prostaglandin receptors coupled to Gα_q_ may be also involved in the regulation of PKC, receptors for other classes of inflammatory mediators, such as chemokines, may also be involved. Future studies are needed to address these issues.

### Concluding remarks

In this report, we have demonstrated that cultured H9c2 cardiomyocytes are capable of initiating a robust inflammatory response in the setting of LPS stimulation. This response involves p38 MAPK and the MAPK negative regulator Mkp-1. We also provide strong evidence implicating crosstalk between p38 MAPK and GPCRs mediated by COX-2/PGE_2_. Since G-proteins are involved in a variety of physiological processes, and many drugs targeting GPCRs have been developed, it is plausible that existing G-protein-targeting drugs may have additional applications in the treatment of cardiac dysfunction in patients with sepsis. In this regard, understanding the role of G-proteins, MAPKs, and their downstream mediators in the pathogenesis of septic shock and cardiovascular dysfunction may reveal novel treatment options for this devastating disease.
